# Analysis of the osseointegrative force of a hyperhydrophilic and nanostructured surface refinement for TPS surfaces in a gap healing model with the Göttingen minipig

**DOI:** 10.1186/s13018-016-0434-6

**Published:** 2016-10-17

**Authors:** Roland Seidling, Lars J. Lehmann, Manuel Lingner, Eckhard Mauermann, Udo Obertacke, Markus L. R. Schwarz

**Affiliations:** 1 Department for Experimental Orthopaedics and Trauma Surgery, Orthopaedic and Trauma Surgery Centre (OUZ), University Medical Centre Mannheim, Medical Faculty Mannheim, Heidelberg University, Theodor-Kutzer-Ufer 1-3, 68167 Mannheim, Germany; 2 Department of Anesthesia and Intensive Care Medicine, Asklepios Südpfalzklinik Kandel, Luitpoldstr. 14, 76870 Kandel, Germany; 3 Clinic of Trauma and Hand Surgery, Vincentius-Kliniken gAG Karlsruhe, Südendstr. 32, 76137 Karlsruhe, Germany; 4 Clinic for Anaesthesia, Intensive Care and Pain Therapy, BG Trauma Centre, Ludwig-Guttmann-Str. 13, 67071 Ludwigshafen, Germany; 5 Department for Anesthesia, Surgical Intensive Care, Prehospital Emergency Medicine and Pain Therapy, University Hospital Basel, Spitalstrasse 21, 4031 Basel, Switzerland

**Keywords:** Osseointegration, TPS, Hyperhydrophilicity, Nanostructure, Gap model, Minipig

## Abstract

**Background:**

A lot of advantages can result in a high wettability as well as a nanostructure at a titanium surface on bone implants. Thus, the aim of this study was to evaluate the osseointegrative potential of a titan plasma-sprayed (TPS) surface refinement by acid-etching with chromosulfuric acid. This results in a hyperhydrophilic surface with a nanostructure and an extreme high wetting rate.

**Methods:**

In total, 72 dumbbell shape titan implants were inserted in the spongy bone of the femora of 18 Göttingen minipigs in a conservative gap model. Thirty-six titan implants were coated with a standard TPS surface and 36 with the hyperhydrophilic chromosulfuric acid (CSA) surface. After a healing period of 4, 8, and 12 weeks, the animals were killed. The chronological healing process was histomorphometrically analyzed.

**Results:**

The de novo bone formation, represented by the bone area (BA), is increased by approximately 1.5 times after 12 weeks with little additional benefit by use of the CSA surface. The bone-to-implant contact (BIC), which represents osseoconductive forces, shows results with a highly increased osteoid production in the CSA implants beginning at 8 and 12 weeks compared to TPS. This culminates in a 17-fold increase in BIC after a healing period of 12 weeks. After 4 weeks, significantly more osteoid was seen in the gap as de novo formation in the CSA group (*p* = 0.0062). Osteoid was also found more frequently after 12 weeks at the CSA-treated surface (*p* = 0.0355). The site of implantation, intertrochanteric or intercondylar, may influence on the de novo bone formation in the gap.

**Conclusions:**

There is a benefit by the CSA surface treatment of the TPS layer for osseointegration over an observation time up to 12 weeks. Significant differences were able to be shown in two direct comparisons between the CSA and the TPS surface for osteoid formation in the gap model. Further trials may reveal the benefit of the CSA treatment of the TPS layer involving mechanical tests if possible.

**Electronic supplementary material:**

The online version of this article (doi:10.1186/s13018-016-0434-6) contains supplementary material, which is available to authorized users.

## Background

According to Albrektsson et al., the implant surface quality is one of six factors that influence the biological response of a bone to an implant [1]. That is the reason why today, the entire sectors of research are engaged in various surface modifications. In these branches, the important keywords are “nanostructure” and “wettability” among many others. Both should lead to an improved and accelerated osseointegration.

Nanoscale modification seems to be a very broad and promising field. It can alter the chemistry and/or topography of the implant surface, and cell culture studies reveal that a range of nanoscale topography exists that promotes the osteoinductive molecular program for adherent osteoprogenitor cells [2]. This nanoscale topography can stimulated macrophages [3] and provoke a certain kind of reaction in many other cells [4].

The wettability of titan surfaces is another subject of research. You can almost postulate that a surface with a high wettability generally improves the osseointegrative process. In vitro, a high wettability of a surface leads to a clear reduction in cell number, but there is an increased expression of osseophilic genes [5–8]. In vivo, you can see an increased bone-to-implant contact (BIC) and an improved mechanical stability at the beginning of the ingrowth process [9–14]. Some examples show that hydrophilic surface refinements lose their force of wettability when exposed to atmospheric air due to the oxidation or adhesion of hydrocarbons [8, 15–18]. Therefore, a correct storage of the implants until implantation is important. In dental implantology, implants devices with a hyperhydrophilic surface have been in use for several years and show good long-term results.

In orthopedics, one main cause of implant failures is the aseptic loosening [19] presumable triggered by debris originating from the articulation components [20, 21]. Thus, debris are a focus of research [22]. At the side of the implant components, which come in contact with the bone, an early and tight anchorage may protect against failure due to debris in the first time after implantation by the so-called sealed interface [23] of the gaps between implant and bone as described by Schmalzried et al. [24] as “effective joint space”. This space may have a higher impact in revision surgery when bone loss occurred due to the excision of the former implant. Thus, the implant surfaces should be able to induce filling up gaps, with the demand for adequate models for preclinical test procedures. Long-term results are hard to evaluate with animal models in the research of joint replacement as we expect life spans of several years or even decades. However, early and tight osseointegration is one of the most important features of an implant surface, which might be evaluable within several weeks after implantation in preclinical tests.

In orthopedics, uncemented implantation systems may have the advantage in the case of revision in comparison to cemented implants because none or at least fewer bone and no bone cement has to be removed. Other orthopedic replacement systems wear an additional layer, for example CaP, to enhance the bone ingrowth [25]. Further developments lead to resorbable CaP coating which enhances osseointegration in the first time after implantation, thus avoiding additional interfaces as the layer is gone after some weeks [26].

As far as we know, no titan-surface with a nanostructure and a high wettability is yet being used in orthopedic joint replacement. The aim of this study was to examine the influence on the osseointegration of a porous titan plasma-sprayed (TPS) implant surface after an etching procedure with chromosulfuric acid [27–30]. After this procedure, the chromosulfuric acid (CSA) surface exhibits a nanostructure as well as a very high wettability [27–30]. In an in vivo model, gaps between implant and bone served as region of interest (ROI) in terms of osseoconductivity and osseoinductivity [31] over a healing period from, respectively, 4, 8, and 12 weeks.

## Methods

### The implants

**Fig. 1:**
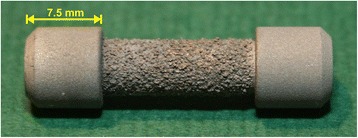
Original CSA implant with the typical dumbbell shape configuration leading to a circular gap around the middle thinner part when inserted across a bone. The fixation in the bone is achieved by anchoring the outer parts in the cortical bone initially by the press-fit technique subsequently by osseointegration

For implantation, 72 non-functional, cylindrical dumbbell shaped implants (length 30 mm) (Fig. 1) were machined from TiAl6V4. They consisted of cylindrical ends for fixation and a bar (15 mm) that enclosed the part of the ROI. This bar was coated with a normal TPS layer [32] with a thickness of approximately 310 μm, measured on a representing implant by the manufacturer (DOT, Rostock, Germany).

The working group of Prof. Jennissen, University Clinics Essen, University of Duisburg-Essen, Germany, and the Fa. Morphoplant GmbH, Bochum, Germany, completed the modification of the implants.

**Fig. 2:**
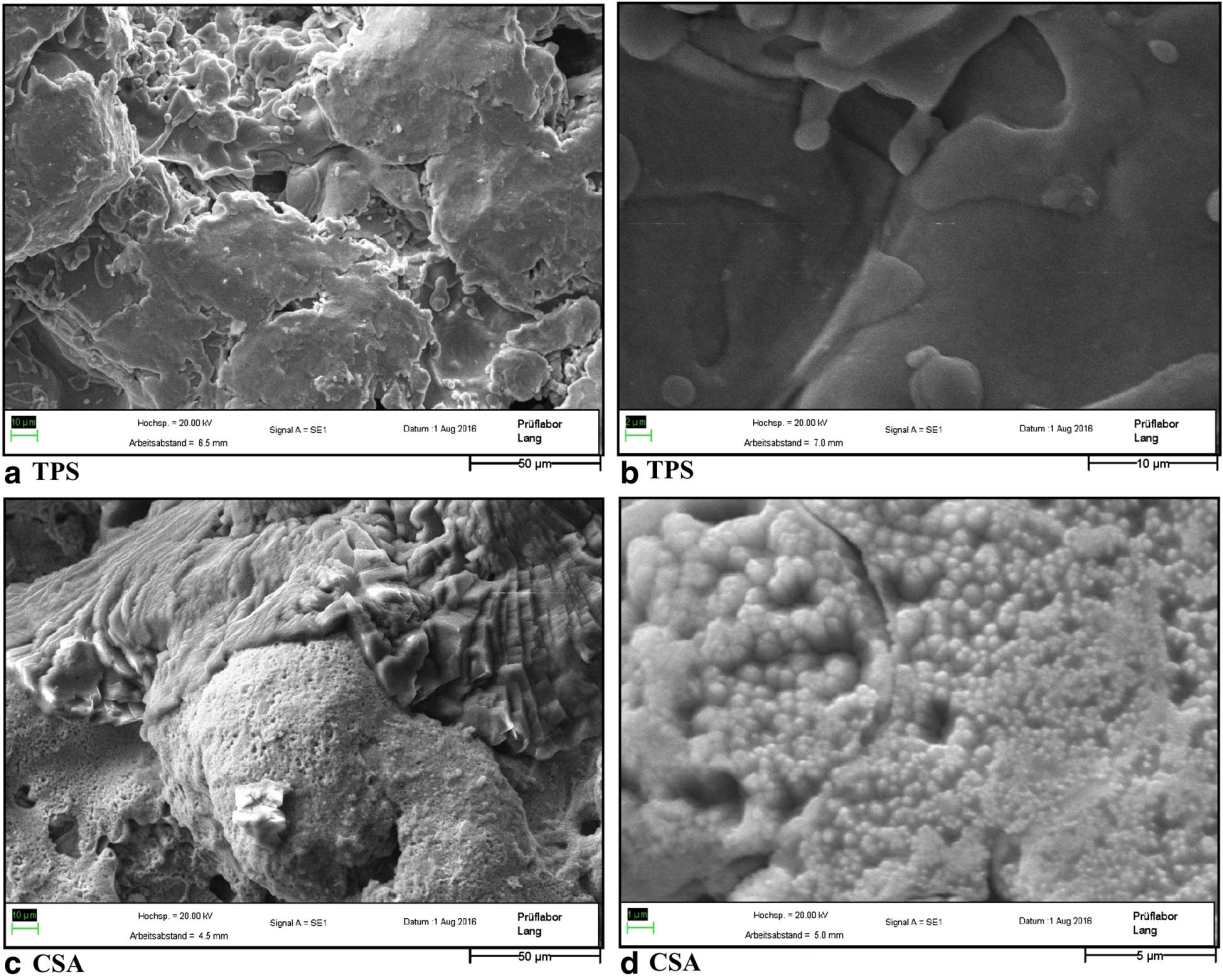
SEM images of the surfaces in different magnifications taken 9 years after manufacturing the implants. **a**, **b** The TPS surface. In both magnifications, there are structures reminding of molten metal splashes. **c** On microscale level, the CSA-treated TPS surface has different forms arguably depending on the orientation of the crystal structure. **d** In a higher magnification, spherical patterns appear with dimension at the nanoscale level. (With permission from Labor Lang, Nürnberg, Germany)

Thirty-six implants had an untreated TPS surface. See Fig. 2 for a SEM image. After cleaning them ultrasonically in 80 % ethanol solution and gamma sterilization (25 kGy), the implants were ready for use. Another 36 other implants were surface enhanced with concentrated CSA by the temperature jump method [27–30]. According to the supplier information [17, 27, 30], the inner bars of the implants should exhibit a TPS surface with a nanostructure and an extremely high wettability after this procedure and sterilization (Fig. 2). Because of the extensive tests of the physical features and the stable etching procedure performed by [16, 17, 27–30, 33–35], we did not carry out further tests of the implants.

### Animals and groups

The breeding company Ellegaard, Dalmose, Denmark, supplied us with 18 skeletally mature, female Göttingen minipigs (GMP). The mean age of the animals at the date of the operation was 45 months (standard deviation (SD) ±6.66, min. 35, max. 57, median 42), and the mean weight was 62 kg (SD ±5.25, min. 54, max. 73, median 62). After biometrical sample size estimations, we formed two groups consisting of nine GMPs each: a control group with 36 TPS implants and an experimental group with 36 CSA implants. Each group was divided in three subgroups corresponding to their postoperative observation time of 4, 8, and 12 weeks, respectively. In all subgroups, each GMP received four implants of the same type according to Schwarz et al. [26].

We chose a period of 4 to 12 weeks. The European norm for the biological evaluation of medical devices (EN 30993-6) requires 3 months for long-term testing.

### Surgery and implantation

The surgery occurred under general anesthesia according to Lehmann et al. and Schwarz et al. [36, 37]. This procedure was very similar to the breeder’s recommendations [38]. But there is a huge wealth in general anesthesia and in operational procedures of the GMPs, [26, 36–40] are only few examples of methodical procedures.

The operational process was carried out as described by Schwarz et al. [26]. Thomsen et al. already described a similar approach for hip arthroplasty in the GMPs in 1997 [41].

**Fig. 3:**
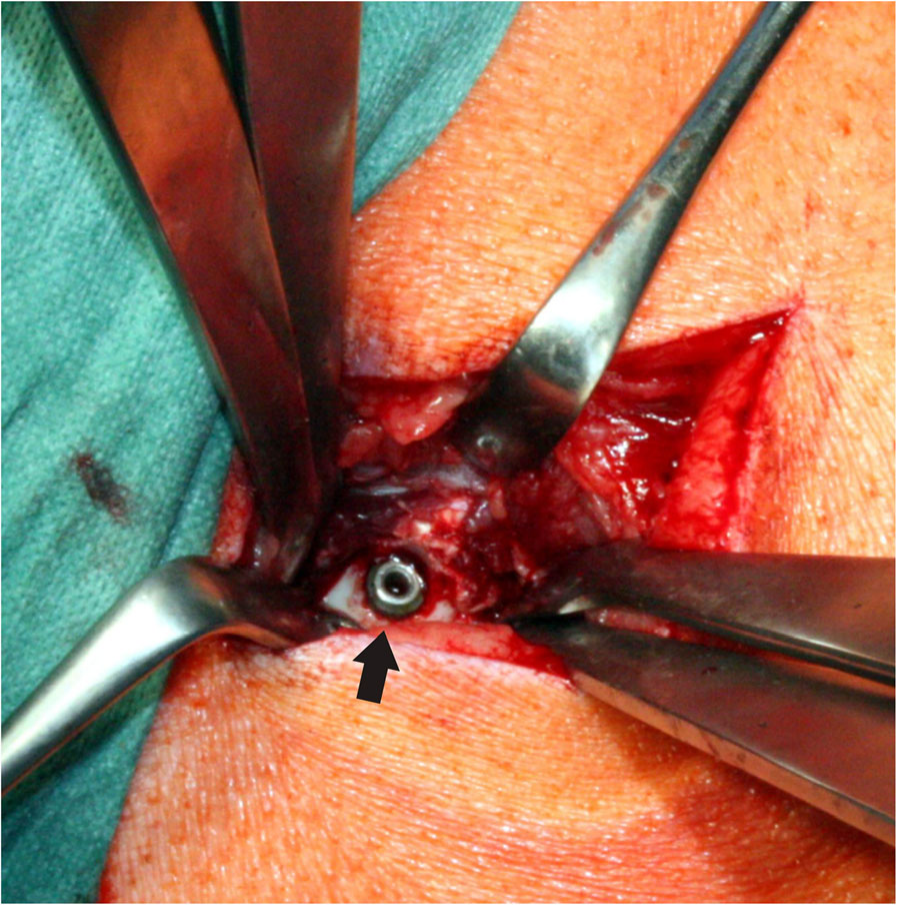
The operation wound which is widened by retractors. The insertion was performed from the lateral site. The implant with a diameter of approximately 8 mm (*arrow*) is placed according to the measured depth of the bore; thus, both ends of the implant will be surrounded by cortical bone by the press-fit technique

**Fig. 4:**
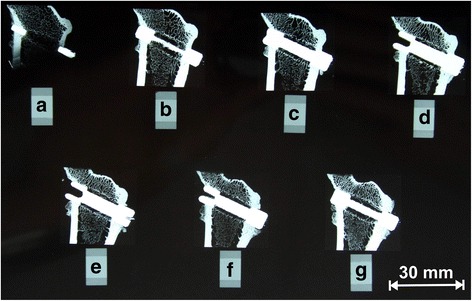
The sections were x-rayed (20 kvp and 5 s) after the preparation of the MCP grinding machine in a Faxitron (Faxitron X-Ray Corporation, Oregon, USA) in a contact X-ray technique. The figure shows a radiograph X-ray of the slices of the CSA preparation of the implant number 901 with the surrounding bony tissue. Seven sections were able to be made (**a**–**g**), and the most middle (**d** and **e**) were taken for further preparation for histology and fluoroscopy. This implant lies in the metaphyseal part of the femur close to the hip

Two implants were inserted in the metaphyseal part of each femur, one in the hip area (intertrochanteric) and one in the knee area (intercondylar). Following the exact measurement of the maximal outer diameter of the implants, we drilled holes that were exactly 0.1 mm smaller than the measurements. This ensured a press-fit implantation [26, 39] (Figs. 3 and 4). In combination with the special design of the implant, this procedure produces an approximately 1-mm circumferential gap around the bar of the implant which was designed to be filled by bone structures during the healing period.

### Sample preparation

After the GMP was killed we removed the femora and the soft tissue around it. At the same time, we checked for changes indicating inflammation and other irritation and also verified the stability of the implants. The diaphyses of the femora were cut orthogonally to its axis leaving a safety distance of 1.3 cm between the cut and the implant. The preparation for histological examination followed the description of Donath and Breuer [42] for non-decalcified specimens. Water and fat were eliminated over a period of some weeks through an ascending ethanol sequence starting at 40 % and ending at 100 %. This process was completed using xylene. Afterwards, the specimens were plasticized using polymethyl methacrylate (PMMA, Fa. Merck, Darmstadt, Germany). Once they had completely hardened, the PMMA blocks were trimmed down from a size of approximately 10 × 10 × 10 cm blocks to a size of 3 × 3 × 3 cm blocks with a special cutting system (Type 36/94, Fa. ﻿EXAKT, Norderstedt, Germany). The smaller PMMA blocks were then cut into fine sections using a minimal contact point cutting system (MCP, ﻿Fa. EXAKT, Norderstedt, Germany).

**Fig. 5:**
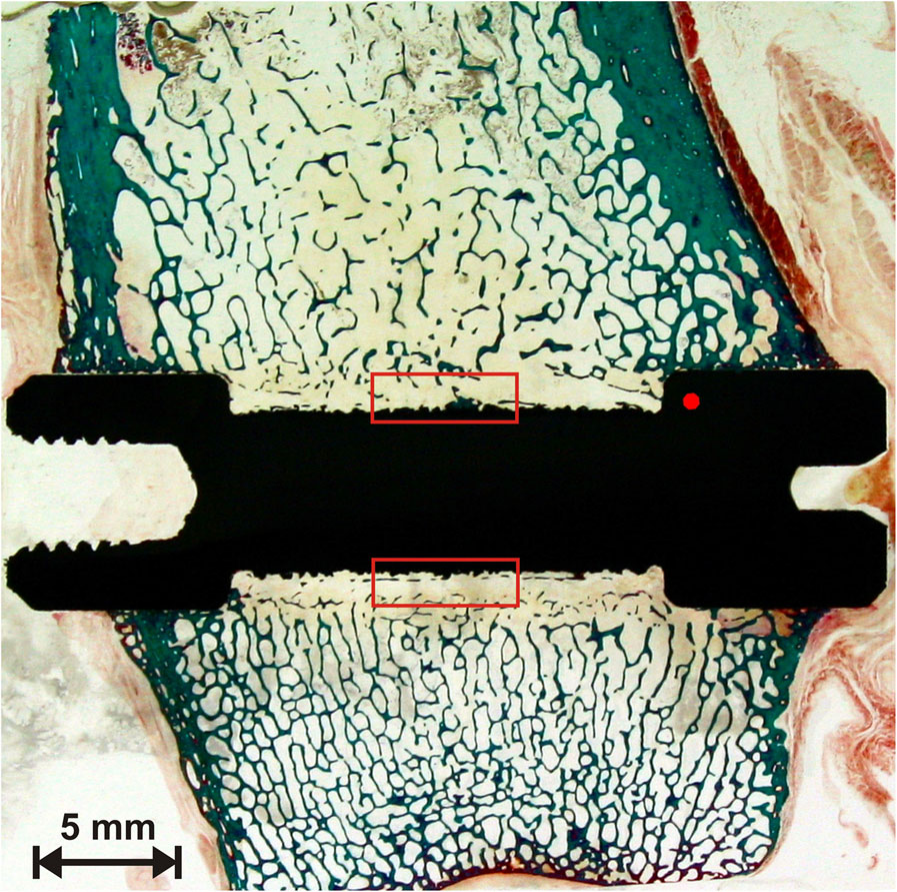
Histological specimen (Masson-Goldner staining) of a CSA implant (preparation 107) with the marked ROIs in the middle of each gap (*red boxes*). This area was measured with a semiautonomously working analysis program. The *red dot* marks the medial head at the diaphyseal side of the implant. The implantation side was the intercondylar part of the left knee. Note the demarcation of the gap also after a healing period of 12 weeks

During the cutting process, two reference points were used for adjustment: the weight bearing axis of the diaphysis and the axis of the implants itself. This way, sections with an orientation strictly parallel to both axis were created (Fig. 4). The two slices closest to the implant axis were glued to slides and grinded down to a thickness level of approximately 100 μm using a special grinding system (400CS, Fa. ﻿EXAKT,﻿﻿ Norderstedt, Germany). Of each pair, the slices closest to the axis was stained by use of a modified Masson-Goldner staining technique [43] (Fig. 5), which allowed us to differentiate between mature bone and osteoid, representing the precursor of bone. The other slice was evaluated using fluorescence microscopy.

### Histomorphometrical evaluation

**Fig. 6:**
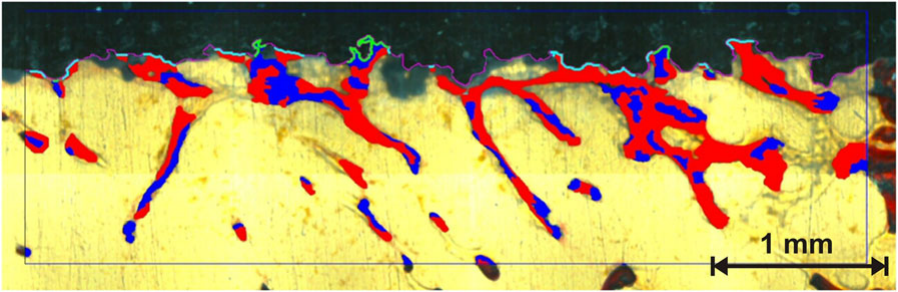
The picture itself is made from 126 single pics to guarantee a high resolution. As example, it shows an augmented detail of the left distal knee gap of a CSA implant (preparation # 55) after a healing period of 8 weeks. The *blue box* marks the ROI (equal to the red box of Fig. 4), and the *pink line* shows the porous CSA surface. The *dark blue* regions demonstrate the bone, and the *red* regions the osteoid areas. The *light green lines* mark the cross section between bone and CSA surface; equal to this, the *turquoise lines* show the cross section for osteoid

All slices were blinded, so that an attribution to an individual group of implants was impossible. The criterions of exclusion for the histological preparations were defined, and pictures of the remaining gaps were taken using a LEICA DRC 300 FX microscope (Fa.﻿ LEICA Camera AG, Solms, Germany). In the pictures, certain zones were highlighted with the cursor: the ROI measuring 5 mm in width and centered in the middle of the gap, the border of the porous TPS /CSA surface as active area for osseoconduction, and the osteoid and the bone area, respectively (Fig. 6). From these data, all information was automatically calculated using the QWIN software (Fa. LEICA Camera AG, Solms, Germany). The results were automatically rendered as percentage relative to its parameter of reference. Each gap was evaluated by three independent observers, and the means were calculated for further use in the statistical analysis. For self supervision, one histological specimen was chosen and the observer repeated the measurements of this one specimen after every 20th gap. The results were then compared to prevent an intraobserver failure.

### Intravital staining

**Fig. 7:**
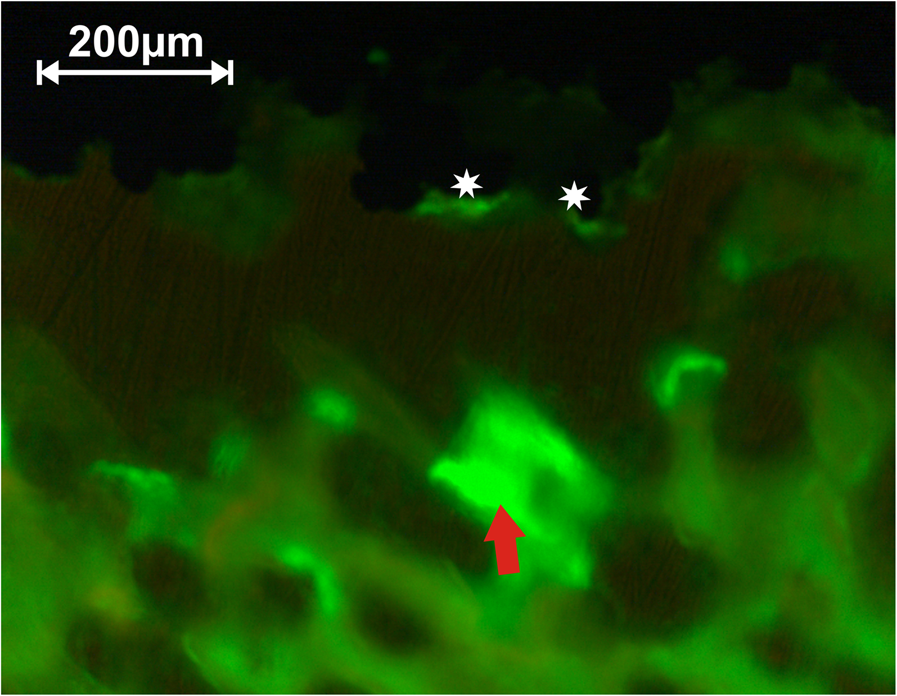
After illumination of the ROI with a wavelength of 370 nm, the fluorescence of tetracycline in the bone matrix is shown. The *arrow* marks a “very high” intensity, “5” as numerical value. The slice of preparation 133 belongs to a CSA implant after 12 weeks of healing, so that the intensity can be seen as activity of bone maturation at the 65th postoperative day. The *stars* mark the implant surface which is here exemplarily in contact to the bone matrix

During the postoperative weeks, an intravital staining was performed [43]. Each GMP received three scheduled intramuscular injections: tetracycline (26 mg/kg BW, Fa. Pfizer GmbH, Karlsruhe, Germany) 19 days (Fig. 7), xylenol orange (90 mg/kg BW, Fa. Waldeck GmbH & Co. KG, Münster, Germany) 12 days, and calcein green (20 mg/kg BW, Fa. Waldeck GmbH & Co. KG, Münster, Germany) 4 days prior to the killing. By excitation with light of the corresponding wave lengths, the three observers evaluated these slices in a semiquantitative way. For the overview the so-called general score (min. 0 to max. 3), for the BA the “amount score” (min. 0 to max. 3), and for bone turnover the “intensity score” (min. 0 to max. 5) were used.

Afterwards, the numerical mean of each of the three scores was calculated and a descriptive statistic was performed.

### Statistical methods

All statistical calculations were made under the guidance of the Department of Statistics and Empirical Research of the Medical Faculty of Mannheim of the University of Heidelberg using SAS 9.4. The level of significance was 0.05.

Based on an estimated relative difference of 15 %, the sample size calculation with a power of 80 % led to a sample size of 11 implants. Taking into account the animal model, the number of implants per subgroup was rounded up to 12 implants. Thus, three animals per subgroup were needed. In total, nine animals received 36 CSA and nine animals 36 TPS implants.

Basically, each implant delivered two gaps, which could possibly be evaluated. The mean of the proximal and the distal gap was calculated; if only one gap was measurable, single values were used for further statistical processing. If both gaps had to be excluded, the implant was lost for the statistical evaluation.

The means of measurements of the three independent observers of the parameters osteoid ongrowth and BIC in percent, as well as osteoid volume and BA in percent, display the four dependent variables of this trial. Before further processing, a Kolmogorov-Smirnov test has shown that the data are not normally distributed and a Levene’s test missed the homoscedasticity of the data. Consecutively, the results of the four parameters were logarhythmated after the addition of 1 (BA, osteoid volume) or 10 (BIC, osteoid ongrowth), respectively, before further processing. Using the new values of the four parameters, a three-way ANOVA with the “PROC MIXED Approach” for repeated measures analysis [44] was calculated. After this, pairwise comparisons between CSA and TPS were made in a two-way ANOVA with the same SAS approach for a healing period of 4, 8, and 12 weeks.

The different values of the length of the implant surface were taken as absolute values of the measurement in millimeters. Here, after a Kolmogorov-Smirnov-test for normal distribution, a one-way ANOVA with a Bonferroni-adjustment was calculated.

## Results

The 18 GMPs survived the operation procedures and were killed on the scheduled days. There were no obvious complications during or after the operation procedures like heavy bleedings, bone fracture, or infection of the implant site. All animals were able to walk without an apparent handicap at the latest 5 days after operation so that the surgical model as well as the pain management appeared to be effective and sufficient.

Several gaps or whole implants had to be excluded, and a total of 93 out of the 144 gaps were evaluated. The gaps were excluded for the following reasons: malpositioning of the implant with no standardized cover of the gaps by bone (*n* = 24), delamination of the TPS layer from the substrate (*n* = 24) and mistakes in histological preparation (*n* = 3). In total, the criterions of exclusion lead to five lost CSA and two lost TPS implants.

After the measurements, the calculated gap height of the CSA implants were as follows: 1.26 mm, SD ±0.07 mm, min. 1.08 mm, max. 1.46 mm, median 1.26 mm; and for the TPS implants: mean 1.28 mm, SD ±0.05 mm, min. 1.21 mm, max. 1.45 mm, median 1.28 mm.

The “length” of the porous surface in the ROI for CSA was 9.34 mm with an SD of ±1.42 mm (min. 7.18 mm, max. 13.26 mm, median 9.01 mm). In the TPS group, the values were as follows: mean 10.57 mm, SD ±1.33 mm, min. 8.31 mm, max. 14.50, and median 10.60 mm, which was significantly different (*p* < 0.0001) from the results of the CSA group.

### Statistical results overall

The rawdata of the histomorphometric analyses is provided as Additional file 1. Perusing the results of the ANOVA overall, the *p* values (Table 1) show that significant differences between the means of CSA and TPS can be found in the data stock for osteoid ongrowth, the BIC, and for osteoid volume. The implantation site (location) shows significances for the BA (Table 1).

**Table 1:** The calculated *p* values of the three-way ANOVA using the “MIXED Procedure” of SAS

	Osteoid ongrowth	BIC	Osteoid volume	BA
Implant type	0.0039	0.0411	0.0327	0.2951
Postoperative time	0.0698	0.1418	0.7630	0.8434
Location	0.1267	0.2080	0.1269	0.0025
Implant type × postoperative time	0.2139	0.2310	0.7908	0.8516
Implant type × location	0.7979	0.5282	0.7572	0.0130

Corresponding to the level of significance of 0.05, several significant differences are found

In the further course, the results of the post hoc tests of the interaction *implant type* and *postoperative time* deliver a more precise explanation of the osseointegration for the corresponding parameters (Table 2).

**Table 2:** The table displays the means and standard deviation of the pairwise comparisons

Parameters	Healing period (weeks)	CSA	TPS	*p* values
Osteoid ongrowth	4	1.32 % ±1.94	0.15 % ±0.26	0.0859
	8	8.67 % ±10.26	0.46 % ±1.08	0.1051
	12	8.10 % ±5.39	1.18 % ±1.74	0.0355
BIC	4	0.85 % ±1.83	0.01 % ±0.03	0.1953
	8	2.47 % ±4.03	0.53 % ±1.34	0.4694
	12	9.00 % ±8.58	0.52 % ±0.83	0.1154
Osteoid volume	4	4.98 % ±1.96	1.89 % ±0.85	0.0062
	8	4.51 % ±2.88	1.97 % ±1.70	0.2691
	12	3.16 % ±1.88	2.41 % ±2.25	0.5014
BA	4	7.83 % ±5.60	7.20 % ±4.51	0.7026
	8	8.55 % ±5.21	6.61 % ±3.58	0.5530
	12	8.78 % ±5.92	5.93 % ±4.25	0.4700

Values are shown in percentages to the corresponding parameters as well as the standard deviation in percentages. *P* values are calculated in a two-way ANOVA using the “MIXED Procedure” of SAS

### Results of the osseoconduction

**Fig. 8:**
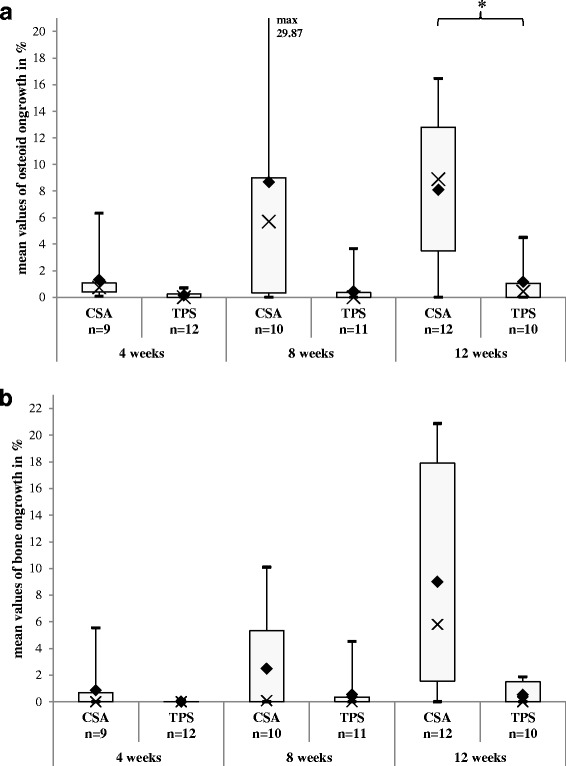
**a** Osteoid ongrowth: In the ANOVA, a *p* value of 0.2139 (Table 1) was calculated for the interaction implant type and postoperative time. The *rhombi* mark the means, and the *crosses* the medians of each group. The *boxes* display the interquartile range. After 12 weeks of defect healing, you can find a significant difference (*p = 0.0355) (Table 2). **b** BIC: A *p* value of 0.2310 (Table 1) was calculated in the ANOVA. In the pairwise comparison, you can find no significant difference between CSA and TPS (Table 2). But after a healing period of 12 weeks, the amount of BIC is 17-fold increased at the CSA surface

The ANOVA revealed two significant results for osteoid ongrowth (*p* = 0.0039) and the BIC (*p* = 0.0411). The osseoconduction is determined by the contact between the porous implant surface and the osteoid or the bone, in our experiment called “osteoid ongrowth” (Fig. 8a) and the “BIC” (Fig. 8b). Although a significant difference is expected for both parameters, only for the osteoid ongrowth a significant difference between CSA and TPS could be shown in the pairwise comparisons of the corresponding healing period.

After 4 weeks of healing, 1.32 % (SD ±1.89) of the CSA surface is in contact to the osteoid. In the TPS group, there is a coverage of 0.15 % (SD ±0.26). After 8 weeks, the difference between CSA and TPS spreads but without a significance: CSA (8.67 %, SD ±10.26) and TPS (0.46 %, SD ±1.08). After 12 weeks of defect healing, this difference (CSA, 8.10 %, SD ±5.39/TPS, 1.18 %, SD ±1.74) shows a significance with *p* = 0.0355.

For the BIC, the means are as follows: after a healing period of 4 weeks, there is an increased amount of bone in contact with the CSA surface (0.85 %, SD ±1.83) in comparison to TPS (0.01 %, SD ±0.03). In both groups, both these values increase, but much faster for CSA (8 weeks, 2.47 %, SD ±4.03/12 weeks, 9.00 %, SD ±8.58) than in the TPS group (8 weeks, 0.53 %, SD ±1.34/12 weeks, 0.52 %, SD ±0.83). After 12 weeks, the amount of BIC is 17-fold increased on the CSA surface in comparison to TPS. But this difference failed to show a significant difference.

### Results of the de novo bone formation

**Fig. 9:**
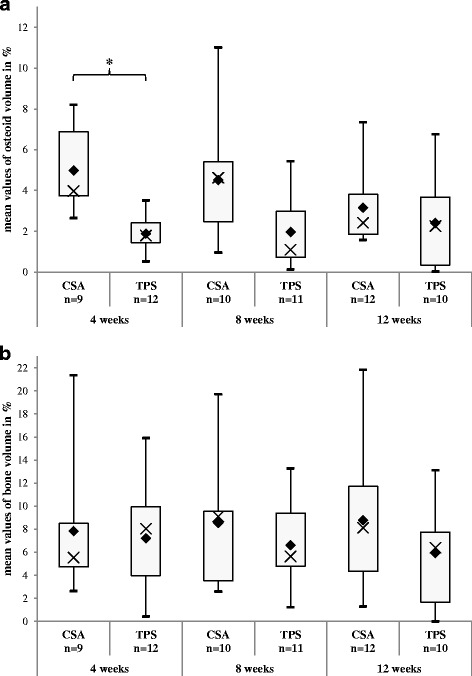
**a** Osteoid volume: In the ANOVA, a *p* value of 0.7908 (Table 1) was calculated for the interaction implant type and postoperative time. The *rhombi* mark the means, and the *crosses* the medians of each group. The *boxes* display the interquartile range. At 4 weeks, you can find a significant difference in the post hoc test between CSA and TPS (* *p* = 0.0062) (Table 2). **b** Bone area: For this parameter, no significant differences could be shown, neither in the ANOVA (*p* = 0.8516) (Table 1) nor in the pairwise comparison (Table 2)

The calculated *p* values of the ANOVA of the de novo bone formation are *p* = 0.0327 for the osteoid (Fig. 9a) and *p* = 0.2951 for the BA (Fig. 9b). After a healing period of 4 weeks, there is a significantly increased (*p* = 0.0062) amount of osteoid for CSA (4.98 %, SD ±1.96) in comparison to TPS (1.89 %, SD ±0.85). These initial high values for CSA decrease about 40 % at 8 weeks (4.51 %, SD ±2.88) to 12 weeks (3.16 %, SD ±1.88). In contrast, the osteoid volume for TPS increases slowly (8 weeks, 1.97 %, SD ±1.70/12 weeks, 2.41 %, SD ±2.25).

While the BA for CSA remains nearly constant (4 weeks, 7.83 %, SD ±5.60/8 weeks, 8.55 %, SD ±5.21/12 weeks, 8.78 %, SD ±5.92), it decreases slowly for TPS (4 weeks, 7.20 %, SD ±4.51/8 weeks, 6.61 %, SD ±3.58/12 weeks, 5.93 %, SD ±4.25). Here, no significant difference could be shown.

### Intravital staining

**Fig. 10:**
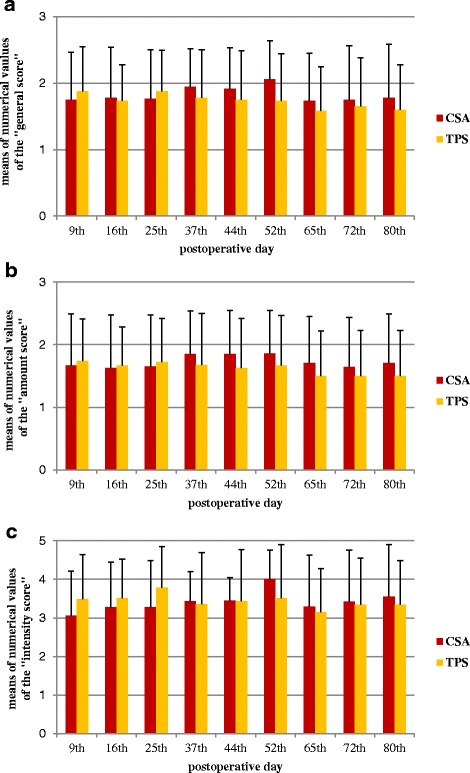
Intravital staining: The figure displays the three evaluated scores. **a** The “general score,” **b** the “amount score,” and **c** the “intensity score”. They should give an overview of the bone formation by the fluorescence microscopy as a semiquantitative evaluation. The injections were performed continuously, so that the whole research period is represented, described by the postoperative days. The *error bars* display the SD

The results of the intravital staining demonstrate that bone turnover took place in the gaps. We were able to see a positive reaction resulting from the integration of the pigments in the bone matrix during the calcification for each date of assessment. Thus, bone metabolism in the gap was proven for every implant till the end of the trial at 12 weeks in similar degrees (Fig. 10).

## Discussion

The aim of the study was the investigation of the osseointegration of a newly created nanostructured and hyperhydrophilic surface refinement for TPS surfaces. The acceleration of osseointegration is the goal of several previous and may be future studies, as there is a growing need for revision surgery as a necessary way of treatment. In revision surgery, gaps between bone and implants will occur more frequently than in primary surgery as the bone stock is reduced depending on, for example, the loosening of the implant or a previous implantation with bone cement. However, also in primary implantation of artificial joints, early bone integration may induce longer survival as the “effective joint space” [24] will be closed early and sealed. Thus, the investigation assessment of CSA surfaces on titanium implants in a gap model seemed to be justified.

### Methods

In comparison to human conditions, the GMP as a large laboratory animal for research offers a great similarity in metabolism, bone density, mineralization, and recovery rate [41, 45]. In this animal, the general anesthesia [38, 40] as well as some operation procedures are well-established. In the present study, a solid model was used, which could be validated for press-fit implantation of cylindrical implants and converted into a gap model as described previously [39]. Holes in cortical bone greater than 400 μm are primarily filled with woven bone [46]. In animals, the inflammatory phase takes place during the first few days, and the reparative phase with woven bone occurs after 2 weeks [25]. Afterwards, a reorganization takes place and a lamellar bone with an functional orientation is formed [25]. The chosen gap width of approximately 1 mm is close to the critical gap size, which shows a delayed bridging by woven bone [25] referred to [47].

This way and by reducing the size of the ROI to 5 mm, representing one third of the total gap length, we were able to evaluate the de novo formation of bone (BA) in a conservative model. This formation was not influenced by regenerating processes based on existing bone at the edge of the gap. Sumner et al. [48] postulated that a bioactive surface (recombinant bone morphogenetic protein) could induce an increased osseointegration on a non-bioactive surface which is implanted in the same animal but in a different location. This effect seemed to be independent of the regenerative ability of the bone morphogenetic proteins but also affected by factors resulting from the regenerating bone around the bioactive surface. To prevent such an effect from happening, every experimental animal in our study received one type of implant. Although this procedure has advantages, it could lead to other problems. If a noticeable osseointegrative reaction in a certain animal took place, it is allocated to either the test or the control group. Thus, a single animal could become a source of inaccuracy. Alternatively, when test and control implants are placed in the same experimental animal, an outlier would be allocated to both groups. Thus, it would not influence the pairs made in the evaluation.

The calculated gap height after the measurements is very comparable in both groups without a significant difference. Assigning these results to the corresponding surfaces, hardly any difference between both could be found (CSA 1.26 mm, SD ±0.07/TPS 1.28 mm, SD ±0.05). Thus, the standardized position of the ROI is justified and the used gap model can be seen as stable with comparable conditions for both groups.

Another interesting parameter is the “length” of the porous surface in the ROI. This parameter was primarily used as a reference for the calculation of the ongrowth. The comparison of the groups revealed a very interesting fact: the means of the CSA (9.34 mm, SD ±1.42 mm) are about 1.2 mm shorter than in the TPS group (10.57 mm, SD ±1.33 mm), with *p* < 0.0001 a significant difference. These measurements could lead to the assumption that the CSA etching may induce a material-consuming process which destroys matter and could end up in a reduction of the surface area on a microscale level. But it has to be considered that the etching with chromosulfuric acid produces a characteristic surface configuration on a nanoscale level leading to an enhancement of the surface area [30] by spheroidic bubbles at nanometer scale in diameter (Fig. 2).

The histomorphometry is still seen as the “golden standard” for the evaluation of undecalcified slices of bone [49, 50]. By the use of the Masson-Goldner staining, a differentiation between osteoid and bone is possible. In combination with the timeframe (4, 8, 12 weeks), the chronological process of the osseointegration is evident. All measurements on the slices are two-dimensional, but in reality, the parameters exhibit three dimensions. But this is a common procedure in histomorphometry and the results have a high correlation to 3D [51, 52].

Theoretically, the intravital staining should give additional information of the dynamic process of osseointegration. Because of the semiquantitative scores, the data does not have the validity of an exact histomorphometrical analysis. But it shows that bone turn over took place over the assessed period.

### Criterions of exclusion

The criterions of exclusion for the slices were rigorous to obtain standardized gaps. Only slices with a detectable successful press-fit implantation and a straight border of spongy bone were evaluated. If a gap was positioned directly next to the cortical bone, it was excluded because here, an increased bone formation into the gap could be assumed. In some slides, the TPS and the CSA surfaces were peeled off the TiAl6V4 body material. Later on, a CSA and TPS implant was checked exemplarily by the manufacturer microscopically. According to this data, one may assume that both the CSA and the TPS surfaces have an adhesion strength of at least 40 MPa [32] on the TiAl6V4 base material. This power could not be in a regeneration process. It must be assumed that this phenomenon arises during the cutting process using the MCP machine. Perhaps in several cases, there was a correlation between an increased noise and vibration and emitted sparks. Thus, thermic damage of the porous surface as well as the surrounding PMMA matrix cannot be excluded. A force-induced delamination in vivo is not likely as the implant is without function in this model. The grinding procedure for thinning the slides for the histological analysis can also weaken the interface between the TPS layer and the substrate as mentioned by the manufacturer [53]. The intended sample size of 11 was reduced to 9 evaluable implants for CSA-4-weeks and to 10 for CSA-8-weeks and TPS-12-weeks. In the other three cases, all implants were usable for examination.

### Histological data

A CSA preparation on a sandblasted-large-grit titanium surface was tested in a pilot study by [30, 54] with a healing period of 4 weeks in dog mandibels. The relative bone density in a distance from <1 mm and >1 mm around the implants was increased in both cases by approximately 100 % [30, 54].

As shown in Fig. 8a, b, the CSA surface shows an osseoconductive property throughout the study. On closer inspection, the amount of the osteoid ongrowth (CSA) remains static from the 8th week on. In a synopsis with the progression of the BIC, it seems that between week 8 and week 12, a high calcification process begins leading to a BIC 17 times higher at the CSA surface after a healing period of 12 weeks in comparison to TPS. A significant difference could only be shown for the osteoid ongrowth after a healing period of 12 weeks (*p* = 0.0355). At first sight, the means seem to display a serious advantage by the CSA surface. At second sight, the further statistical parameter show some “positive” outliers and a broad variance of both ongrowth parameters.

Figure 9a, b shows the chronological process of the de novo bone formation with less difference in the pairwise comparisons than in the ongrowth parameters. Here, fast CSA-induced osteoid formation shows significance after 4 weeks of healing before it starts to decrease till the 12th week. But at this time, the osteoid volume of CSA is still 1.3 times higher than in the TPS group. The bone area (BA) for CSA is increasing constantly while the values decrease for the TPS. After 12 weeks, the BA for CSA is 1.5 times higher, but without significance.

A similar regeneration behavior is reported in the same gap model. For a CaP-enhanced surface, the BIC shows an increase after 4 (12.5 %) and 8 weeks (32 %) while for TPS, the values stays low (0.01 % at 4 and 0.7 % at 8 weeks). For the BA, hardly any difference could be detected [39].

In the ANOVA the term “location” (*p* = 0.0025) and the interaction “implant type × location” (*p* = 0.0130) of the bone volume gives an indication for significant differences, each time with an increased bone formation at the intertrochanteric (hip) insertion point in comparison to the intercondylar (knee). Maybe their different bone stocks provide different osseointegrative potential. Because this phenomenon is not reproducible for the other three parameters, the discussion of this aspect will be postponed.

One surface refinement similar to the CSA etching is the sandblasted, large-grit, and acid-etched (SLA)-processing. These implants exhibit a microrough surface [55] and after a correct storage a low DCA of approximately 0° [56] and show an accelerated BIC formation [9, 12] with an increase between 1.2 to 1.5 times in comparison to a conventional storage of SLA. In comparison to Jennissen and Lüers [17] and Alfarsi et al. [56] SLA displays comparable surface properties to TPS, with regard to roughness and wettability. In a removal torque test, little difference could be noticed between the SLA- and the TPS surface [57]. So the difference in BIC is approximately 17 times higher in the CSA group compared to the TPS group, which is remarkable in the presented study. This is perhaps due to the high wetting rate [33] in the composition of the nanostructures and needs further study.

A positive effect of the CSA surface on the osseointegration can be seen over the assessed time period up to 12 weeks. However, only 2 of 12 pairwise comparisons between the TPS and the CSA groups showed significant differences for both parameters: the osteoid ongrowth and the osteoid volume. At first sight, the ongrowth parameters seem to display a serious advantage on the osseointegration. At second sight, the statistical parameters show some “positive” outliers and a broad variance of both ongrowth parameters. It seems that the CSA surface as well as the TPS surface have no manifest ability of bridging a gap near the critical gap size of 1 mm [25] referred to [47] in the assessed healing period up to 12 weeks. For a hydroxyapatite coating, a sufficient sealing effect could be shown from 3 weeks after surgery [58, 59]. However the use of a titan monolayer surface eliminates the risk of the destruction of an additional layer, as described by Soballe as the “degradability in biological environment” [25].

For a regular TPS surface, a BIC of approximately 22 % after a press-fit implantation in a Göttingen minipig could be shown [26]. Despite of the data of the gap model, we suggest that results received from mechanical testing of the CSA surface could generate very interesting results, because the surface could develop its osseoconductive potential when it makes direct contact to the existing bone. This could be analyzed in a press-fit model with pull-out tests as described previously [26] or by the use of screw shape implants with removal torque tests [60, 61].

## Conclusions

The presented study leads to the conclusion that there is a benefit by the CSA surface treatment of the TPS layer for osseointegration over an observation time up to 12 weeks. Statistical proof was able to be shown in two direct comparisons between the CSA and the TPS surface in the gap model in terms of osteoid.

Further trials may reveal the benefit of the CSA treatment of the TPS layer particularly involving mechanical tests if possible.

## Additional file

Additional file 1: Supplemental material. (XLSX 62 kb)

## Abbreviations

BA: Bone area
BIC: Bone-to-implant contact
CSA: Chromosulfuric acid
DCA: Dynamic contact angle
GMP: Göttingen minipig
MCP: Minimal contact point
MMA/PMMA: (poly-)methylmethacrylat
ROI: Region of interest
SD: Standard deviation
SLA: Sandblasted, large grit, and acid-etched
TPS: Titan plasma spray

## References

[1] Albrektsson T (1981). Osseointegrated titanium implants. Requirements for ensuring a long-lasting, direct bone-to-implant anchorage in man. Acta Orthop Scand.

[2] Mendonça G (2008). Advancing dental implant surface technology—from micron- to nanotopography. Biomaterials.

[3] Chen S (2010). Characterization of topographical effects on macrophage behavior in a foreign body response model. Biomaterials.

[4] Bettinger CJ, Langer R, Borenstein JT (2009). Engineering substrate topography at the micro- and nanoscale to control cell function. Angew Chem Int Ed Engl.

[5] Klein MO (2010). Long-term response of osteogenic cells on micron and submicron-scale-structured hydrophilic titanium surfaces: sequence of cell proliferation and cell differentiation. Clin Oral Implants Res.

[6] Klein MO (2013). Submicron scale-structured hydrophilic titanium surfaces promote early osteogenic gene response for cell adhesion and cell differentiation. Clin Implant Dent Relat Res.

[7] Masaki C (2005). Effects of implant surface microtopography on osteoblast gene expression. Clin Oral Implants Res.

[8] Olivares-Navarrete R (2012). Osteoblast maturation and new bone formation in response to titanium implant surface features are reduced with age. J Bone Miner Res.

[9] Abdel-Haq J (2011). Osseointegration and stability of a modified sand-blasted acid-etched implant: an experimental pilot study in sheep. Clin Oral Implants Res.

[10] Bornstein MM (2008). Bone apposition around two different sandblasted and acid-etched titanium implant surfaces: a histomorphometric study in canine mandibles. Clin Oral Implants Res.

[11] Jimbo R (2013). Histomorphometry and bone mechanical property evolution around different implant systems at early healing stages: an experimental study in dogs. Implant Dent.

[12] Lang NP (2011). Early osseointegration to hydrophilic and hydrophobic implant surfaces in humans. Clin Oral Implants Res.

[13] Park JW, Kwon TG, Suh JY (2013). The relative effect of surface strontium chemistry and super-hydrophilicity on the early osseointegration of moderately rough titanium surface in the rabbit femur. Clin Oral Implants Res.

[14] Vasak C (2013). Early bone apposition to hydrophilic and hydrophobic titanium implant surfaces: a histologic and histomorphometric study in minipigs. Clin Oral Implants Res.

[15] Park JH (2012). The responses to surface wettability gradients induced by chitosan nanofilms on microtextured titanium mediated by specific integrin receptors. Biomaterials.

[16] Jennissen HP (2010). Stabilizing ultra-hydrophilic surfaces by an exsiccation layer of salts and implications of the Hofmeistereffect. Stabilisierung ultra-hydrophiler Oberflächen durch eine Exsikkationsschicht aus Salzen und Bedeutung des Hofmeister-Effektes. Mater Werkst.

[17] Jennissen HP, Lüers S (2010). Lotus-effect and inverse Lotus-effect in connection with extremely rough titanium surfaces. Lotus- und inverser Lotus-Effekt im Zusammenhang mit extrem rauen Titanoberflächen. Mater Werkst.

[18] Straumann® Dental Implant System (2011). Straumann® SLActive WISSENSCHAFTLICHE STUDIEN FÜNFTE AUSGABE.

[19] Sadoghi P (2013). Revision surgery after total joint arthroplasty: a complication-based analysis using worldwide arthroplasty registers. J Arthroplasty.

[20] Purdue PE (2006). The central role of wear debris in periprosthetic osteolysis. HSS J.

[21] Harris WH (2001). Wear and periprosthetic osteolysis: the problem. Clin Orthop Relat Res.

[22] Schroder C (2013). Characterization of polyethylene wear particle: the impact of methodology. Acta Biomater.

[23] Sundfeldt M (2006). Aseptic loosening, not only a question of wear: a review of different theories. Acta Orthop.

[24] Schmalzried TP, Jasty M, Harris WH (1992). Periprosthetic bone loss in total hip arthroplasty. Polyethylene wear debris and the concept of the effective joint space. J Bone Joint Surg Am.

[25] Soballe K (1993). Hydroxyapatite ceramic coating for bone implant fixation. Mechanical and histological studies in dogs. Acta Orthop Scand Suppl.

[26] Schwarz ML (2009). Effect of surface roughness, porosity, and a resorbable calcium phosphate coating on osseointegration of titanium in a minipig model. J Biomed Mater Res A.

[27] Jennissen HP (2001). Ultrahydrophile metallische Oberflächen. Biomaterialien.

[28] Jennissen HP (2005). Ultra-hydrophilic transition metals as histophilic biomaterials. Macromol Symp.

[29] Jennissen HP (1999). Biocoating of implants with mediator molecules: surface enhancement of metals by treatment with chromosulfuric acid. Mater Werkst.

[30] Lattner D, Jennissen HP (2009). Preparation and properties of ultra-hydrophilic surfaces on titanium and steel. Mater Werkst.

[31] Albrektsson T, Johansson C (2001). Osteoinduction, osteoconduction and osseointegration. Eur Spine J.

[32] DOT GmbH, Charles-Darwin-Ring 1a, 18059 Rostock, Deutschland: Schichtdossier TPS. 2016

[33] Jennissen HP (2012). Hyperhydrophilic rough surfaces and imaginary contact angles Hyperhydrophile raue Oberflächen und imaginäre Kontaktwinkel. Mater Werkst.

[34] Jennissen HP (2014). A general mathematical form and description of contact angles. Mater Werkst.

[35] Jennissen HP, Luers S, Laub M. Hyperhydrophilic surfaces, the inverse lotus effect and imaginary contact angles*.* Biomed Tech. 2012:s1-D.

[36] Lehmann LJ (2011). Scintigraphic evaluation of rhBMP-2-biocoated implants reveals no ectopic bone formation. Biomed Pharmacother.

[37] Schwarz M (2010). Scintigraphic evaluation of bone formation in Göttinger minipigs. Scand J Lab Anim Sci.

[38] Alstrup AK (2010). Anaesthesia and analgesia in Ellegaard Göttingen minipigs.

[39] Schwarz M, Feuerstack M, Herbig J, Brade J, Becker K, Scheller G. Effect of a resorbable cap coating on bone-implant contact and density in a gap model after 4 and 8 weeks. An experimental study in Göttinger minipigs. 52th Annual Meeting of the Orthopaedic Research society. March 19-22, 2006, Chicago Illinois, USA, 2006.

[40] Ettrup KS et al. Basic surgical techniques in the Gottingen minipig: intubation, bladder catheterization, femoral vessel catheterization, and transcardial perfusion. J Vis Exp. 2011;26(52). 10.3791/2652 PMC3197034 21730947

[41] Thomsen M (1997). The Gottinger minipig as an animal model in hip endoprosthesis. Anatomy, anesthesia, operation results. Z Orthop Ihre Grenzgeb.

[42] Donath K, Breuner G (1982). A method for the study of undecalcified bones and teeth with attached soft tissues. The Sage-Schliff (sawing and grinding) technique. J Oral Pathol.

[43] Romeis B, Plenk H jr. Untersuchung des Binde- und Stützgewebes In: Böck P, editor. Mikroskopische Techniken. 17th ed. München-Wien-Baltimore: Urban & Schwarzenberg; 1989. p. 491–566.

[44] Walker GA, Shostak J. Common statistical methods for clinical research with SAS examples, third edition, vol. chapter 8. Cary: SAS Institue Inc; 2010. p. 136–137. ISBN 978-1-60764-228-2.

[45] Pearce AI (2007). Animal models for implant biomaterial research in bone: a review. Eur Cell Mater.

[46] Johner R. Dependence of bone healing on defect size. Helv Chir Acta. 1972;39(1):409–11. 5034289

[47] Schenk R, Lane JM (1987). Cytodynamics and histodynamics of primary bone repair. Fracture healing.

[48] Sumner DR (2004). Locally delivered rhBMP-2 enhances bone ingrowth and gap healing in a canine model. J Orthop Res.

[49] Iwaniec UT, Wronski TJ, Turner RT (2008). Histological analysis of bone. Methods Mol Biol.

[50] Yeom H (2008). Correlation between micro-computed tomography and histomorphometry for assessment of new bone formation in a calvarial experimental model. J Craniofac Surg.

[51] Lahm A (2014). Correlation between 3D microstructural and 2D histomorphometric properties of subchondral bone with healthy and degenerative cartilage of the knee joint. Histol Histopathol.

[52] Stadlinger B (2013). Osseointegration of biochemically modified implants in an osteoporosis rodent model. Eur Cell Mater.

[53] DOT GmbH, Charles-Darwin-Ring 1a, 18059 Rostock, Deutschland: Universitätsmedizin Mannheim - Beurteilung der TPS-Grenzschicht. Projektauswertung F026-16, 2016

[54] Becker J (2006). Bone apposition to titanium implants biocoated with recombinant human bone morphogenetic protein-2 (rhBMP-2). A pilot study in dogs. Clin Oral Investig.

[55] Le Guehennec L (2008). Histomorphometric analysis of the osseointegration of four different implant surfaces in the femoral epiphyses of rabbits. Clin Oral Implants Res.

[56] Alfarsi MA, Hamlet SM, Ivanovski S (2013). Titanium surface hydrophilicity modulates the human macrophage inflammatory cytokine response. J Biomed Mater Res A.

[57] Novaes AB (2004). Influence of implant microstructure on the osseointegration of immediate implants placed in periodontally infected sites. A histomorphometric study in dogs. Clin Oral Implants Res.

[58] Rahbek O (2000). Sealing effect of hydroxyapatite coating: a 12-month study in canines. Acta Orthop Scand.

[59] Rahbek O (2005). Superior sealing effect of hydroxyapatite in porous-coated implants: experimental studies on the migration of polyethylene particles around stable and unstable implants in dogs. Acta Orthop.

[60] Gotfredsen K, Berglundh T, Lindhe J (2000). Anchorage of titanium implants with different surface characteristics: an experimental study in rabbits. Clin Implant Dent Relat Res.

[61] Gotfredsen K (1995). Anchorage of TiO2-blasted, HA-coated, and machined implants: an experimental study with rabbits. J Biomed Mater Res.

